# Is chronic inhibition of phosphodiesterase type 5 cardioprotective and safe? A meta-analysis of randomized controlled trials

**DOI:** 10.1186/s12916-014-0185-3

**Published:** 2014-10-20

**Authors:** Elisa Giannetta, Tiziana Feola, Daniele Gianfrilli, Riccardo Pofi, Valentina Dall’Armi, Roberto Badagliacca, Federica Barbagallo, Andrea Lenzi, Andrea M Isidori

**Affiliations:** Department of Experimental Medicine, Sapienza University of Rome, Viale del Policlinico 155, Rome, 00161 Italy; Unit of Clinical and Molecular Epidemiology, IRCCS San Raffaele Pisana of Rome, Via della Pisana 235, Rome, 00163 Italy; Department of Cardiovascular and Respiratory Science, Sapienza University of Rome, Piazzale Aldo Moro 5, Rome, 00185 Italy

**Keywords:** Phosphodiesterase-5, Heart failure, Hypertension, Endothelial function, Cardiac remodeling, Cardiac hypertrophy, Erectile dysfunction, Sildenafil, Pulmonary hypertension

## Abstract

**Background:**

The myocardial effects of phosphodiesterase type 5 inhibitors (PDE5i) have recently received consideration in several preclinical studies. The risk/benefit ratio in humans remains unclear.

**Methods:**

We performed a meta-analysis of randomized, placebo-controlled trials (RCTs) to evaluate the efficacy and safety of PDE5i on cardiac morphology and function. From March 2012 to December 2013 (update: May 2014), we searched English-language studies from MEDLINE, EMBASE, Cochrane Central Register of Controlled Trials and SCOPUS-selecting RCTs of continuous PDE5i administration that reported cardiovascular outcomes: cardiac geometry and performance, afterload, endothelial function and safety. The pooled estimate of a weighted mean difference between treatment and placebo was obtained for all outcomes using a random effects model. A test for heterogeneity was performed and the I^2^ statistic calculated.

**Results:**

Overall, 1,622 subjects were treated, with 954 randomized to PDE5i and 772 to placebo in 24 RCTs. According to our analysis, sustained PDE5 inhibition produced: (1) an anti-remodeling effect by reducing cardiac mass (−12.21 g/m^2^, 95% confidence interval (CI): −18.85; −5.57) in subjects with left ventricular hypertrophy (LVH) and by increasing end-diastolic volume (5.00 mL/m^2^; 95% CI: 3.29; 6.71) in non-LVH patients; (2) an improvement in cardiac performance by increasing cardiac index (0.30 L/min/m^2^, 95% CI: 0.202; 0.406) and ejection fraction (3.56%, 95% CI: 1.79; 5.33). These effects are parallel to a decline of N-terminal-pro brain natriuretic peptide (NT-proBNP) in subjects with severe LVH (−486.7 pg/ml, 95% CI: −712; -261). PDE5i administration also produced: (3) no changes in afterload parameters and (4) an improvement in flow-mediated vasodilation (3.31%, 95% CI: 0.53; 6.08). Flushing, headache, epistaxis and gastric symptoms were the commonest side effects.

**Conclusions:**

This meta-analysis suggests for the first time that PDE5i have anti-remodeling properties and improve cardiac inotropism, independently of afterload changes, with a good safety profile. Given the reproducibility of the findings and tolerability across different populations, PDE5i could be reasonably offered to men with cardiac hypertrophy and early stage heart failure. Given the limited gender data, a larger trial on the sex-specific response to long-term PDE5i treatment is required.

**Electronic supplementary material:**

The online version of this article (doi:10.1186/s12916-014-0185-3) contains supplementary material, which is available to authorized users.

## Background

For many years, the use of selective phosphodiesterase type 5 inhibitors (PDE5i) has been limited to on-demand administration for erectile dysfunction (ED) [1]. These drugs have recently been shown to affect hemodynamics, right heart performance and oxygenation in patients with pulmonary arterial hypertension (PAH) [2-4]. In addition, PDE5i are currently used in continuous administration for rehabilitation of erectile function and relief of lower urinary tract symptoms [5], although their systemic effects and safety have not been formally appraised.

Initial studies on PDE5i investigated their possible use in the symptomatic relief of angina. However, this was soon abandoned due to the risk of coronary steal or hypotension. Nevertheless, the potential role of PDE5i in non-urological fields continued to attract interest [6,7]. In parallel, several preclinical experimental studies demonstrated that PDE5i were beneficial in ischemia/reperfusion injury, myocardial infarction, doxorubicin-induced cardiotoxicity, hypertrophic cardiac remodeling and heart failure (HF) [8,9]. All these conditions share an enhanced expression of PDE5 enzyme in cardiomyocytes.

Despite these promising animal data, the cardioprotective effect of PDE5i in humans remained unclear. Of note is the discrepancy between the amount of solid experimental data and the paucity of translational studies for drugs that are readily available and widely used with other indications. The few human studies primarily targeting specific cardiac conditions - post-myocardial infarction diastolic dysfunction, diabetic cardiomyopathy, mild and severe heart failure [10-13] - suggest a potential effect of PDE5i on cardiac kinetics, geometry and performance, although not universally [14]. Controversy also exists concerning the claimed mechanisms: some hypothesized a direct effect within cardiomyocytes [11,12], some suggested coronary [15] and peripheral vasodilation [16] and yet others a peripheral endothelial effect [17]. However, a much larger number of trials is available reporting cardiac outcomes as secondary endpoints after continuous PDE5i administration.

We performed this meta-analysis to investigate whether chronic PDE5 inhibition modulates cardiac parameters in different clinical settings, exploring both cardiac and peripheral vascular effects, since these endpoints have never been fully assessed in a comprehensive review of the literature.

Specially, we aimed to answer the following questions:

‘Does chronic administration of PDE5i produce clinically meaningful changes in cardiac remodeling and performance?’ , ‘Does chronic administration of PDE5i produce hemodynamic and/or endothelial function changes?’ , ‘Is chronic PDE5i administration well-tolerated and safe?’

## Methods

We performed this study according to the Cochrane Collaboration and PRISMA statement [18].

### Data sources and searches

From March 2012 to December 2013 we searched for English-language articles in MEDLINE, EMBASE, Cochrane Library and SCOPUS. Search terms were: sildenafil/tadalafil/vardenafil/PDE5i AND hypertension/blood pressure/pulmonary hypertension/cardiovascular disease/ heart/heart failure/endothelium/endothelial function. We updated the search in May 2014, but no further studies were included.

### Study selection

Eligibility criteria for study selection included: 1) randomized placebo-controlled trials (RCTs); and 2) chronic PDE5i administration defined as a continuous, daily or alternate day (for tadalafil only based on its half-life), prolonged (≥4 weeks) administration. We selected studies reporting any cardiovascular outcomes (as primary or secondary endpoint) independently of the baseline characteristics of the study population.

We excluded reviews, editorials, commentaries, letters, non-RCTs, animal studies, co-administration of PDE5i with other therapies (that is, endothelin antagonist or bosentan) or measuring outcomes under stress or under hypoxic conditions.

**Figure 1:**
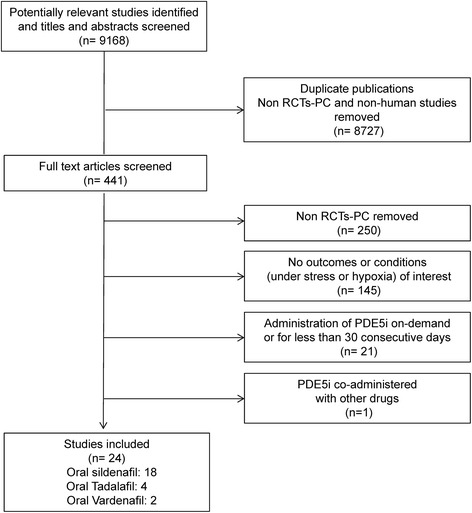
**Study flow diagram.** PDE5i: phosphodiesterase 5-inhibitors; RCTs: randomized placebo-controlled trials.

Three independent reviewers evaluated all selected titles and abstracts, and for articles considered potentially eligible, full text reports were considered. Interobserver agreement was high (98%: 434/441 RCTs chosen for full text analysis). Where disagreement occurred, a unanimous decision was taken after open discussion. Figure 1 shows the literature eligibility assessment process.

### Data extraction and quality assessment

Two reviewers (T.F. and R.P.) independently extracted data on study design, sample population (age, gender, clinical status, comorbidities) and treatment characteristics (active compound: sildenafil, tadalafil, vardenafil; dosage and length of treatment). Table 1 summarizes the features of selected studies [see also Additional file 1: Table S1].

**Table 1:** **Characteristics of studies selected for analysis**

**Study**	**Design**	**Condition**	**Treatment**	**Dose and time**	**Number of patients (PP)**	**Mean age (SD) or age range**
**Aldashev AA,** ***Thorax*** **, 2005** **[** 26 **]**	R, DB, PC	HAPH	S	25 mg x 3/die	9S versus 8P	61 (8)
				100 mg x 3/die 12 weeks	5S versus 8P	
**Amin A,** ***Congest Heart Fail*** **, 2013** **[** 38 **]**	R, DB, PC	HFrEF	S	25 mg x 2/die - >2 weeks	53S versus 51P	S: 51.29 (14.80)
				Then		
				50 mg x 3/die - >10 weeks		P: 50.61 (4.18)
**Andersen MJ,** ***Circulation*** **, 2013** **[** 10 **]**	R, DB, PC	Diastolic Dysfunction after MI	S	40 mg x 3/die 9 weeks	34S versus 33P	S: 63 (8)
						P: 62 (7)
**Badesch,** ***J Rheumatol*** **, 2007** **[** 27 **]**	R, DB, PC	PAH-CTD	S	20 mg x 3/die	21 S	S: 52 (15)
				40 mg x 3/die	20 S	50 (15)
				80 mg x 3/die	19 S	54 (14)
				12 weeks	22 P	P: 56 (14)
**Behling A,** ***J Cardiac Fail*** **, 2008** **[** 23 **]**	R, DB, PC	CHF	S	50 mg x 3/die	11S versus 8P	S: 45 (12)
				4 weeks		P: 53 (11)
**Bharani A,** ***Indian Heart J*** **, 2007** **[** 28 **]**	R, DB, PC	PAH due to congenital left to right shunt	T	20 mg x 3	8 T - P	28 (9.38)
	CO (wo:2 wks)			4 weeks		
**Bocchio M,** ***Atherosclerosis*** **, 2008** **[** 33 **]**	R, DB, PC	ED VRF	T	20 mg every other day	18 T versus 18P	T: 52.05 (8.98)
				12 weeks		P: 49.61 (12.68)
**Galiè N,** ***NEJM*** **, 2005** **[** 2 **]**	R, DB, PC	PAH	S	20 mg x 3/die	65 S	S: 47 (14)
				40 mg x 3/die	63 S	51 (15)
				80 mg x 3/die	65 S	48 (18)
				12 weeks	65 P	P: 49 (17)
**Giannetta E,** ***Circulation*** **, 2012** **[** 11 **]**	R, DB, PC	Diabetic Cardiomyopathy	S	25 + 25+ 50 mg/die	29S versus 25P	S: 60.7 (7.6)
				3 months		P: 60.2 (8.3)
**Goldberg DJ,** ***Circulation*** **, 2011** **[** 36 **]**	R, DB, PC	CHD after Fontan Operation	S	20 mg x 3/die	27 S - P	14.9 (5.1)
	CO (wo:6 wks)			6 weeks		
**Goldberg, DJ** ***Pediatr Cardiol*** **, 2012** **[** 37 **]**	R, DB, PC	CHD after Fontan Operation	S	20 mg x 3/die	27 S - P	14.9 (5.1)
	CO (wo:6 wks)			6 weeks		
**Groeneweg G, BMC** ***Muscoloskeletan disorders*** **, 2008** **[** 20 **]**	R, DB, PC	CRPS	T	10 mg/die - >4 weeks	12 T versus12P	T: 39.8 (13.1)
				Then		P: 36.5 (10.6)
				20 mg/die - >8 weeks		
**Guazzi M,** ***J Am Coll Cardiol*** **, 2007** **[** 24 **]**	R, DB, PC	CHF	S	50 mg x 3/die	23S versus 23P	S: 62 (3)
				6 months		P: 63 (4)
**Guazzi M,** ***Circulation*** **, 2011** **[** 25 **]**	R, DB, PC	HFpEF with PAH	S	50 mg x 3/die	22S versus 22P	53-79
				12 months		
**Guazzi M,** ***Circ Heart Fail,*** **2011** **[** 12 **]**	R, DB, PC	Systolic HF	S	50 mg x 3/die	23S versus 22P	S: 60 (4)
				12 months		P: 61 (4)
**Guazzi M,** ***Europ J Heart*** **Failure, 2012** **[** 39 **]**	R, DB, PC	EOB in HF	S	50 mg x 3/die	16S versus 16P	S: 66 (8)
				12 months		P: 68 (6)
**Jing, Z.C,** ***Am J Resp Crit Care Med*** **, 2011** **[** 30 **]**	R, DB, PC	PAH	V	5 mg/die - >4 weeks	43 V versus 16P	S: 32 (12)
				then		
				5 mg x 2/die - >8 weeks		P: 29 (8)
**Lewis GD,** ***Circulation*** **, 2007** **[** 13 **]**	R, DB, PC	Systolic HF and secondary PAH	S	25 to 75 mg x 3/die	17S versus 17P	S: 54 (4)
				12 weeks		P: 62 (3)
**Lewis GD,** ***Circ Heart Fail,*** **2008** **[** 31 **]**	R, DB, PC	Systolic HF and secondary PAH	S	25 to 75 mg x 3/die	15S versus 15P	S: 54 (4)
				12 weeks		P: 62 (3)
**Rosano G MC,** ***European Urology*** **, 2005** **[** 34 **]**	R, DB, PC	ED VRF	T	20 mg every other day	16 T versus 16P	65.4 (6.3)
				4 weeks		
**Redfield MM,** ***JAMA,*** **2013** **[** 14 **]**	R, DB, PC	HFpEF	S	20 mg x 3/die - >12 weeks	49S versus 47P^a^	62-77
				then 60 mg x 3/die ->	45S versus 58P^b^	
				12 weeks	95S versus 94P^c^	
**Sastry BKS,** ***JACC,*** **2004** **[** 32 **]**	R, DB, PC	PAH	S	25 to 100 mg x 3/die	20 S - P	16-55
	CO (wo:0)			6 weeks		
**Suntharalingam J,** ***Chest,*** **2008** **[** 40 **]**	R, DB, PC	CTEPH	S	40 mg x 3/die	8S versus 10P	S: 49.9 (13.1)
				12 weeks		P: 60 (14.4)
**Van AH,** ***J Sex Med*** **, 2005** **[** 35 **]**	R, DB, PC	ED	V	5 to 20 mg/die	175 V versus 175P	V:22-76
				12 weeks		P: 22-78

^a^patients for left ventricular mass index (LVMi) and end-diastolic volume index (EDVi); ^b^patients for systolic blood pressure (SBP); ^c^patients for N-terminal-pro brain natriuretic peptide (NT-proBNP). CHD: congenital heart disease; CHF: chronic heart failure; CO: crossover; CRPS: cold complex regional pain syndrome; CTEPH: chronic thromboembolic pulmonary hypertension; DB: double-blind; ED VRF: erectile dysfunction vascular risk factors; EOB: exercise oscillatory breathing; HAPH: high altitude pulmonary hypertension; HF: heart failure; HFpEF: heart failure preserved ejection fraction; HFrEF: heart failure reduced ejection fraction; MI: myocardial infarction; P: placebo; PAH: pulmonary arterial hypertension; PAH-CTD: pulmonary arterial hypertension-connective tissue diseases; PC: placebo-controlled; PP: per protocol analysis; R: randomized; S: sildenafil; T: tadalafil; V: vardenafil; wks: weeks; wo: washout.

In order to compare all studies on the effect of the treatment at the end of the planned therapeutic cycle, we excluded *ad interim* data, and only end-of-treatment values were recorded. The third investigator (E.G.) performed quality control checks on extracted data. Risk of bias for all trials was independently assessed by the investigators, using the Cochrane risk-of-bias algorithm [19] [see Additional file 1: Table S2].

### Outcomes

Selected treatment efficacy outcomes were: cardiac geometry (left ventricular mass index: LVMi, end-diastolic volume index: EDVi, interventricular septum: IVS, ventricular transverse diameter: VTD), cardiac performance (cardiac index; ejection fraction: EF; the ratio of the early -E- to late -A- ventricular filling velocities: E/A ratio), neuroendocrine biomarkers (NT-proBNP) and hemodynamic/endothelial parameters (heart rate: HR, blood pressure: BP, systemic vascular resistance index: SVRi, flow mediated vasodilation: FMD). Information on adverse events was extrapolated and analyzed to investigate treatment safety.

### Data synthesis and analysis

Quantitative data extracted from the papers for all treatment efficacy outcomes were baseline and after treatment/placebo means ± standard deviation (SD). When differences from baseline (means ± SD) were reported, these were also extracted. When summary statistics were not adequately or fully reported (for example, missing pre-post treatment mean difference ± SD on a specific outcome; standard error of an estimated effect and no corresponding SD), these were calculated whenever possible. When baseline levels, post-treatment and/or change from baseline data were missing or inconsistent, the authors of the original papers were contacted in order to obtain the necessary information [see Additional file 1: *Statistical Analysis*]. Several studies (16/24) reported data on mixed populations, male and female; however, only 1 of the 16 contacted authors provided raw data separately [20], thus it was not possible to stratify by gender.

In order to examine the efficacy of PDE5i in the different clinical settings, all retrieved studies were categorized *a priori* according to the following categories: 1) moderate-severe left ventricular hypertrophy (LVH) versus non/mild-LVH (based on LVMi values above or below 131 g/m^2^ [21] and where not available, on NT-proBNP levels above or below 700 pg/mL) [22]; 2) left versus right heart disease; 3) cardiac disease versus non-cardiac disease; 4) HF with reduced EF versus HF with preserved EF; 5) age: younger versus older than 60 years of age; and 6) active compound: sildenafil versus tadalafil versus vardenafil. The same categories were also used for the subgroup analysis. A minimum of two studies were used for subgroup analyses; however, findings stemming from such analyses were interpreted with care. Where a specific subgroup involved a single study, as occurred for HF with preserved EF [14], the analysis was not performed.

Adverse events in the treatment group compared to the placebo group were analyzed by relative risks calculated on the intention-to-treat population. Any adverse event found only in one study was not analyzed [see Additional file 1: *Statistical Analysis*].

A meta-analysis was performed on all outcomes and effect sizes were combined to give a pooled estimate of a weighted mean difference (WMD) between treatment and placebo, the weights being the reciprocals of the variance. A random effects model was fitted. This choice was dictated by the heterogeneity of study characteristics.

The test for heterogeneity was performed and the I^2^ statistic (low = 30%; moderate = 30% to 75%; high ≥75%) calculated. All results are shown in Additional file 1-*Heterogeneity*.

Along with estimation results, forest plots and funnel plots were visually inspected in order to more clearly detect clusters of studies, outlier studies and possible publication biases.

Statistical significance was set at *P* <0.05. The software used for all statistical analysis was STATA/SE V10.

## Results

### Study selection

Figure 1 shows the literature search process in MEDLINE, EMBASE, Cochrane and SCOPUS (March 2012 to December 2013 and updated in May 2014). We identified 9,168 studies as potentially relevant. Of these, 8,727 were excluded based on title and abstract content and 417/441 were excluded after full text analysis due to: non-English language, non-human studies, not RCTs, no outcome of interest, PDE5i co-administered with other drugs. RCTs with PDE5i given on demand or for less than 30 consecutive days were excluded. A total of 24 RCTs were eligible and included in the review (18 administering sildenafil, 4 tadalafil and 2 vardenafil).

### Study characteristics

Table 1 summarizes the 24 reports that met all inclusion criteria. Authors analyzed the effects of continuous PDE5i administration in various cardiac disorders (diastolic dysfunction secondary to myocardial infarction [10], HF [12,14,23-25], PAH [26-32], diabetic cardiomyopathy [11]) and ED [33-35]. One study was performed on a non-cardiological/non-urological condition, enrolling patients with complex regional pain syndrome [20]. Goldberg *et al*. [36,37] and Lewis *et al*. [13,31] reported their results in two separate publications, each addressing different endpoints; thus, the 24 retrieved papers described 22 groups of subjects. For parameters reported twice (mean BP, SVR and HR), data from the most recent publications were used [31,37].

Selected trials gave details of 1,622 subjects, with 954 randomized to PDE5i (694 to sildenafil; 54 to tadalafil; 218 to vardenafil), and 772 to placebo (per protocol (PP) analysis), 55 allocated in cross-over trials. Most studies were performed in North America or Europe, with five in Asia [26,28,30,32,38].

The studies varied in terms of: 1) daily dosage: some administered PDE5i with a titration scheme [20,30,38] or based on individual response [14,32,35]; 2) length of treatment: from 4-week to 12-month study periods; 3) endpoint assessment method; 4) age; 5) baseline cardiovascular status; and 6) gender: 8 trials enrolled only males [11,12,24,26,33-35,39], and 16 trials a mixed population of 540 females and 459 males (ITT population). All studies were RCTs, double-blind (DB) and placebo-controlled. Four studies were crossover with variable washout periods (from 0 to 6 weeks) [28,32,36,37].

Around half (56%) of the trials reported cardiovascular data as secondary outcomes. Fourteen trials received funding from pharmaceutical companies (Pfizer, Eli Lilly) [2,13,20,23,26,30,31,34] or foundations [12,25,36,37,39].

### Risk of bias

All publications reported results from RCTs; however, the randomization method and allocation concealment were inappropriately described in 54% of studies (13/24), so the risk of selection bias was unclear for this group, while for the remaining 46% of trials the risk of selection bias was low.

Regarding the reporting bias, seven studies had high risk and in two additional studies it was unclear. Most studies had a low risk of performance, detection and attrition bias [see Additional file 1: *Risk of bias* and Table S2].

### Synthesis of results

#### Efficacy data: PDE-5 inhibitors *versus* placebo

##### Cardiac geometry

The following parameters were considered measures of cardiac structure: LVMi, EDVi, IVS and VTD.

**Figure 2:**
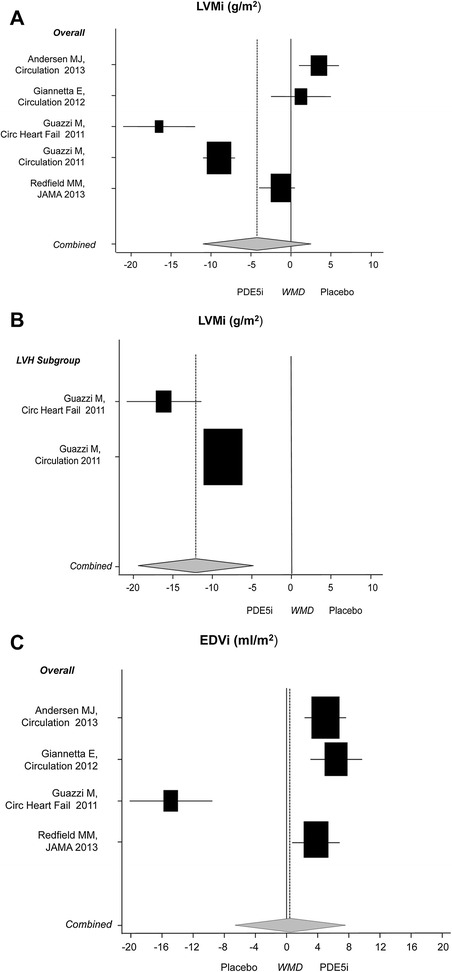
**Effects of PDE5i over placebo on cardiac geometry parameters. A)** LVMi main analysis; **B)** LVMi subgroup analysis in patients with LVH; **C)** EDVi main analysis. Diamond indicates the overall summary estimate for the analysis (width of the diamond represents the 95% CI); boxes, the weight of individual studies in the pooled analysis. EDVi: end-diastolic volume index; LVH: left ventricular hypertrophy; LVMi: left ventricular mass index; PDE5i: phosphodiesterase type 5 inhibitors.

LVMi did not change (−4.022 g/m^2^; 95% CI: −10.137; 2.093, *P* = 0.20) in the main analysis performed in five trials, all using sildenafil [10-12,14,25] (n = 306; 72% male). However, a significant reduction in LVMi (12.207 g/m^2^; 95% CI: −18.846; −5.568, *P* <0.001) was detected in subgroup analysis of patients with left ventricular hypertrophy (Figure 2).

Similarly, the main analysis of EDVi (0.433 mL/m^2^; 95% CI: −6.49; 7.357, *P* = 0.90) in four studies [10-12,14] (n = 262; 71% male) found no significant change. Subgroup analyses revealed a significant increase in EDVi in non-hypertrophic patients (4.999 mL/m^2^; 95% CI: 3.286; 6.711, *P* <0.001, I^2^ = 0.0%) (Figure 2).

The main analysis of IVS [11,25] and VDT [11,12] was possible in two studies only, with no significant change found (Table 2).

**Table 2:** **Results of main analysis and subgroup or sensitivity analyses**

**Main analysis**	**LVMi g/m** ^**2**^	**EDVi ml/m** ^**2**^	**IVS Mm**	**VTD mm**	**Cardiac index L/min/m** ^**2**^	**EF%**	**E/A ratio**	**NTpro-BNP pg/ml**	**HR bpm**	**MAP mmHg**	**SBP mmHg**	**DBP mmHg**	**SVRi dynes m** ^**2**^ **/sec cm** ^**−5**^	**FMD%**
**WMD**	−4.022	0.433	−0.966	−2.666	0.304	3.561	0.053	−161.9	−0.848	−0.413	0.616	−0.763	−88.34	3.306
Lower 95% CI	−10.137	−6.49	−2.230	−7.145	0.202	1.786	−0.275	−351.5	−2.398	−3.023	−1.113	−2.174	−255.75	0.530
Upper 95% CI	2.093	7.357	0.298	1.814	0.406	5.335	0.380	27.6	0.702	2.196	2.345	0.648	79.08	6.082
----	----	----	----	----	----	----	----	----	----	----	----	----	----	---
*Number*	*306*	*262*	*98*	*9*9	520	*286*	*116*	*407*	*977*	*355*	*828*	*791*	*232*	*168*
*P value*	*0.197*	*0.902*	*0.134*	*0.243*	***<0.001***	***<0.001***	*0.753*	*0.094*	*0.283*	*0.756*	*0.490*	*0.289*	*0.301*	***0.020***
I-square	96%	94%	95%	90%	0.0%	84%	93%	82%	56%	71%	49%	52%	89%	99%
References	[10-12,14,25]	[10-12,14]	[11,25]	[11,12]	[2,10,11,25,30,32,40]	[10-12,24,25,31,35]	[12,25,37]	[10-14,40]	[2,10,11,20,24,26,28,30,31,35,37,39]	[10,23,25,30,31,38,39]	[10-12,14,20,24,25,28,35,36,38]	[10-12,20,24-26,28,35,36,38]	[10,25,30,31,39]	[11,24,33,34]
**Subgroup/ Sensitivity** ^**a**^	**LVMi g/m** ^**2**^	**EDVi ml/m** ^**2**^	**IVS Mm**	**VTD mm**	**Cardiac index L/min/m** ^**2**^	**EF %**	**E/A ratio**	**NTpro-BNP Pg/ml**	**HR bpm**	**MAP mmHg**	**SBP mmHg**	**DBP mmHg**	**SVRi dynes m** ^**2**^ **/sec cm** ^**−5**^	**FMD%**
**LVH**	−12.207				0.082	4.382	0.053	−486.7	0.829	1.156	0.135	−1.936	55.63	/
Lower 95% CI	−18.846	/	/	/	−0.176	2.059	−0.275	−712,8	−4.526	−2.398	−2.598	−5.309 1.438	−277.86	
Upper 95% CI	−5.568				0.341	6.705	0.380	−260,7	6.185	4.710	2.867	----	389.12	
---	----				----	----	----	----	----	----	----		----	
*Number*	*89*				*62*	*119*	*116*	*97*	*57*	*74*	*72*	*116*	*74*	
*P value*	***<0.001***	*/*	*/*	*/*	*0.534*	***<0.001***	*0.753*	***<0.001***	*0.761*	*0.524*	*0.923*	*0.261*	*0.744*	*/*
I-square	88%	/	*/*	*/*	0.0%	79%	93%	0.0%	0.0%	0.0%	0.0%	66%	88%	/
**Non-LVH**	1.404	4.999^a^			0.354	0.973		−11.20	0.691		0.613	−0.635	/	/
Lower 95% CI	−1.908	3.286	/	/	0.165	−1.329	/	−160.8	−2.330	/	−5.161	−9.278 8.008		
Upper 95% CI	4.717	6.711			0.544	3.274		138.4	3.713		6.388	----		
----	----	----			----	----		----	----		----			
*Number*	*217*	*217*			*121*	*121*		*310*	*121*		*224*	*121*		
*P value*	*0.406*	***<0.001***	*/*	*/*	***<0.001***	*0.408*	*/*	*0.883*	*0.654*	*/*	*0.835*	*0.886*	*/*	*/*
I-square	80%	0.0%	/	/	0.0%	0.0%	*/*	75%	0.0%	/	79%	91%	/	/
**Left heart disease**	−4.022	0.433	−0.966	−2.666	0.298	3.561	0.053	−149.7^a^	0.651	−0.951	0.079^a^	−1.352	−70.97	2.686
Lower 95% CI	−10.137 2.093	−6.49 7.357	−2.230 0.298	−7.145 1.814	0.134	1.786 5.335	−0.275	−352.4	−2.781 4.084	−4.377 2.474	−2.610 2.768	−3.932 1.227	−273.90	−2.635 8.008
Upper 95% CI					0.461		0.380	53.0					131.95	
----	----	----	----	----	----	----	----	----	----	----	----	----	----	----
*Number*	*306*	*262*	*98*	*9*9	*165*	*286*	*116*	*389*	*229*	*252*	*419*	*360*	*173*	*100*
*P value*	*0.197*	*0.902*	*0.134*	*0.243*	***<0.001***	***<0.001***	*0.753*	*0.148*	*0.710*	*0.586*	*0.954*	*0.304*	*0.493*	*0.322*
I-square	96%	94%	95%	90%	0.0%	84%	93%	84%	81%	75%	66%	75%	91%	99%
**Subgroup/ Sensitivity** ^**a**^	**LVMi g/m** ^**2**^	**EDVi ml/m** ^**2**^	**IVS Mm**	**VTD mm**	**Cardiac index L/min/m** ^**2**^	**EF%**	**E/A ratio**	**NTpro-BNP Pg/ml**	**HR bpm**	**MAP mmHg**	**SBP mmHg**	**DBP mmHg**	**SVRi dynes m** ^**2**^ **/sec cm** ^**−5**^	**FMD%**
**Right heart disease**					0.309				−2.700			0.867		
Lower 95% CI	/	/	/	/	0.178	/	/	/	−4.554	/	/	−2.637 4.372	/	/
Upper 95% CI					0.439				−0.847					
----					----				----			----		
*Number*					*355*				*347*			*30*		
*P value*	*/*	*/*	*/*	*/*	***<0.001***	*/*	*/*	*/*	***0.004***	*/*	*/*	*0.628*	*/*	*/*
I-square	*/*	*/*	*/*	*/*	0.0%	*/*	*/*	*/*	0.0%	*/*	*/*	0.0%	*/*	*/*
**Non-cardiac disease**									−0.109		0.107	0.073		3.953
Lower 95% CI	/	/	/	/	/	/	/	/	−2.723	/	−2.287	−1.515 1.660	/	1.240
Upper 95% CI									2.504		2.502			6.667
----									----		----	----		----
*Number*									*374*		*374*	*374*		*68*
*P value*	*/*	*/*	*/*	*/*	*/*	*/*	*/*	*/*	*0.935*	*/*	*0.930*	*0.928*	*/*	***0.004***
I-square	*/*	*/*	*/*	*/*	*/*	*/*	*/*	*/*	0.0%	/	0.0%	0.0%	/	96%
**Age <60 yrs**					0.284	3.187	0.173^**a**^			1.940	−0.008	−0.292	−130.44	
Lower 95% CI					0.163	−0.716	−0.150			−0.824	−2.023	−1.631 1.047	−234.44	/
Upper 95% CI	/	/	/	/	0.405	7.089	0.496	/	/	4.704	2.006	----	−26.43	
----					----	----	----			----	----	*513*	----	
*Number*					*399*	*74*	*71*			*212*	*513*		*89*	
*P value*	*/*	*/*	*/*	*/*	***0.000***	*0.110*	*0.295*	*/*	*/*	*0.196*	*0.993*	*0.669*	***0.014***	***/***
I-square	/	/	/	/	0.0%	80%	90%	/	/	0.0%	0.0%	0.0%	0.0%	**/**
**Age** **≥** **60 yrs**	−2.683^a^	0.433		−2.666	0.354	2.582		−40.199^**a**^	−0.491	−2.349	0.663	−2.048	−57.48	3.558^a^
Lower 95% CI	−9.169	−6.49	/	−7.145 1.814	0.165	−1.074	/	−196.023	−3.826	−5.851	−2.067	−4.741 0.646	−349.32	0.306
Upper 95% CI	3.804	7.357		----	0.544	6.237		115.624	2.844	1.153	3.392		234.36	6.809
----	----	----			----	----		----	----	----	----	----	----	----
*Nymber*	*262*	*262*		*99*	*121*	*167*		*355*	*221*	*143*	*315*	*234*	*143*	*132*
*P value*	*0.418*	*0.902*	*/*	*0.243*	***<0.001***	*0.166*	*/*	*0.613*	*0.773*	*0.189*	*0.634*	*0.136*	*0.699*	***0.032***
I-square	95%	94%	/	90%	0.0%	90%	/	72%	79%	83%	72.%	71%	94%	99%
**Subgroup/ Sensitivity** ^**a**^	**LVMi g/m** ^**2**^	**EDVi ml/m** ^**2**^	**IVS Mm**	**VTD mm**	**Cardiac index L/min/m** ^**2**^	**EF%**	**E/A ratio**	**NTpro-BNP Pg/ml**	**HR bpm**	**MAP mmHg**	**SBP mmHg**	**DBP mmHg**	**SVRi dynes m** ^**2**^ **/sec cm** ^**−5**^	**FMD%**
**Sildenafil**	−4.022	0.433	−0.966	−2.666	0.289^a^	3.561	0.053	−161.9	−0.943	−0.880^a^	0.248	−0.995	−70.9	2.7
Lower 95% CI	−10.137	−6.49	−2.230	−7.145 1.814	0.179	1.786	−0.275	−351.5	−2.959	−3.642	−2.138	−2.852 0.863	−273.9	−2.6
Upper 95% CI	2.093	7.357	0.298		0.399	5.335	0.380	27.6	1.073	1.882	2.634		131.9	8.0
---	----	----	----	----	----	----	----	----	----	----	----	----	----	----
*Number*	*306*	*262*	*98*	*9*9	*461*	*286*	*116*	*407*	*536*	*296*	*446*	*409*	*173*	*100*
*P value*	*0.197*	*0.902*	*0.134*	*0.243*	***<0.001***	***<0.001***	*0.753*	*0.094*	*0.359*	*0.532*	*0.839*	*0.294*	*0.493*	*0.322*
I-square	96%	94%	95%	90%	0.0%	84%	93%	82%	0.0%	72%	60%	63%	91%	/
**Tadalafil**									−1.033		4.659	1.101		3.953
Lower 95% CI									−7.141		−2.529		/	1.240
Upper 95% CI	/	/	/	/	/	/	/	/	5.075	/	11.846	−4.717 6.920		6.667
---									----		----	----		----
Number									32		32	32		68
P value	/	/	/	/	/	/	/	/	0.740	/	0.204	0.711	/	**0.004**
I-square	/	/	/	/	/	/	/	/	0.0%	/	0.0%	0.0%	/	**/**
**Vardenafil**									−0.408					
Lower 95% CI									−3.746					
Upper 95% CI	/	/	/	/	/	/	/	/	2.931	/	/	/	/	/
---									----					
Number									409					
P value	/	/	/	/	/	/	/	/	0.811	/	/	/	/	/
I-square	/	/	/	/	/	/	/	/	0.0%	/	/	/	/	/

^a^Sensitivity analysis. CI: confidence interval; DBP: diastolic blood pressure; EDVi: end-diastolic volume index; EF: ejection fraction; E/A ratio: the ratio of the early (E) to late (A) ventricular filling velocities; FMD: flow mediated dilation; HR: heart rate; IVS: interventricular septum; LVH: left ventricular hypertrophy; LVMi: left ventricular mass index; MAP: mean arterial blood pressure; NTpro-BNP: N-terminal-pro brain natriuretic peptide; SBP: systolic blood pressure; SVRi: systemic vascular resistance index; VTD: ventricular transverse diameter; WMD: weighted mean difference. Bold indicates when “p” are statistically significant. Italics indicates non significant resluts.

### Cardiac performance

Cardiac index and EF were considered measures of systolic performance and E/A ratio a measure of diastolic function.

**Figure 3:**
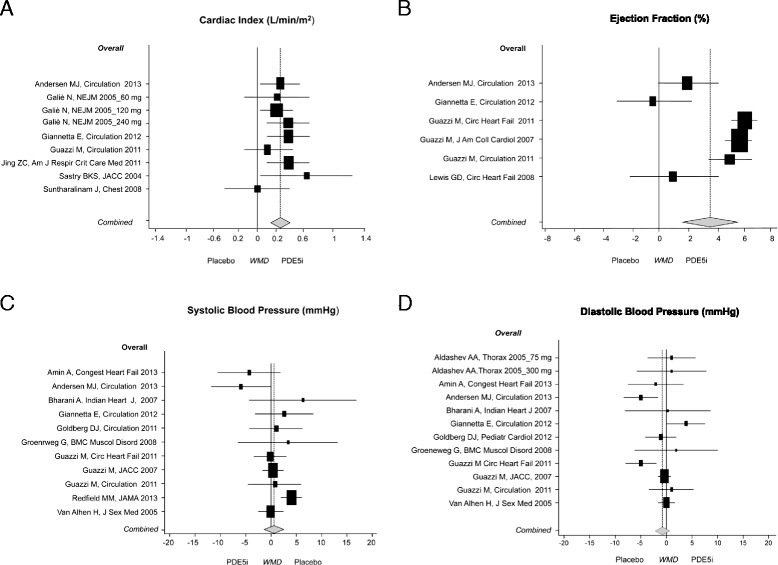
**Effects of PDE5i over placebo on cardiac performance and blood pressure. A)** Main analysis on the cardiac index (L/min/m^2^); **B)** Main analysis on EF (%); **C)** main analysis on SBP (mmHg) and **D)** main analysis on DPB (mmHg). Diamond indicates the overall summary estimate for the analysis (width of the diamond represents the 95% CI); boxes indicate the weight of individual studies in the pooled analysis. CI: confidence interval; PDE5i: phosphodiesterase type 5 inhibitors.

Data on the cardiac index were available in seven studies [2,10,11,25,30,32,40] (520 patients, 65.6% male), showing an increase of 0.304 L/min/m^2^ (95% CI: 0.202; 0.406, *P* <0.001) induced by sildenafil [2,10,11,25,32,40] and vardenafil [30] over placebo [32] (Figure 3). Subgroup analyses confirmed the significant effects on the cardiac index in patients without LVH (0.354 L/min/m^2^; 95% CI: 0.165; 0.544, *P* <0.001) and in patients with left (0.298 L/min/m^2^; 95% CI: 0.134; 0.461, *P* <0.001) or right heart disease (0.309 L/min/m^2^; 95% CI: 0.178; 0.438, *P* <0.001). The PDE5i-dependent increase in the cardiac index was observed in patients both younger and older than the age of 60 (Table 2). These results were confirmed when sensitivity analysis was performed and the study using vardenafil [30] excluded (0.289 L/min/m^2^; 95% CI: 0.179; 0.399, *P* <0.001).

Six studies [10-12,24,25,31] (286 patients, 95.8% male) gave details on EF in patients with left heart disease, in whom sildenafil (n = 146) produced a 3.561% (95% CI: 1.786; 5.335) increase over placebo (n = 140, *P* <0.001) (Figure 3). Subgroup analyses revealed larger effect sizes in patients with LVH (4.382%; 95% CI: 2.059; 6.705, *P* <0.001). The meta-analysis of E/A ratio included three studies [12,25,37] (116 patients, 83.7% male) and showed no significant effect of sildenafil over placebo.

### Neuroendocrine biomarkers

**Figure 4:**
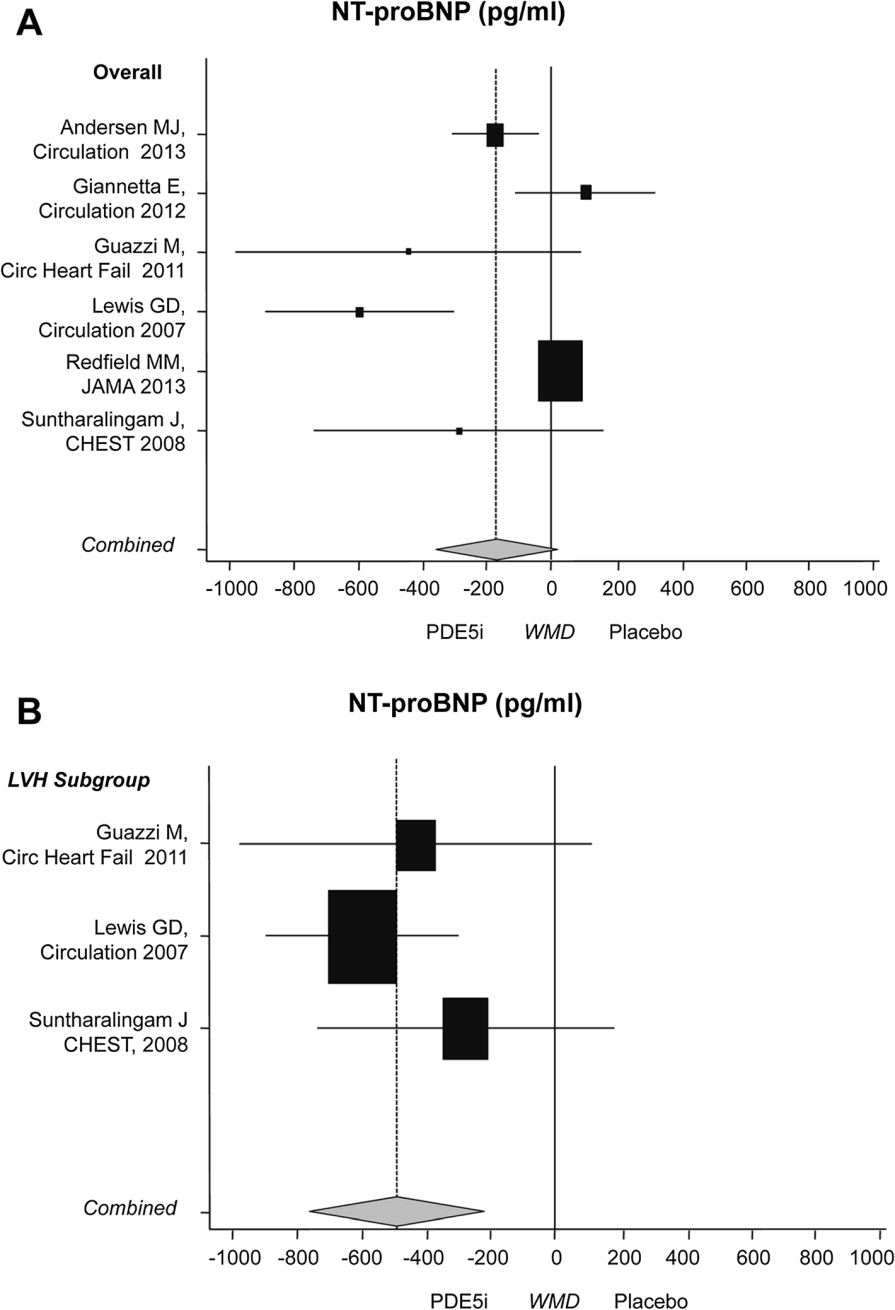
**Effects of PDE5i over placebo on marker of cardiac hypertrophy. A)** NT-proBNP in main and **B)** subgroup analyses for LVH patients. Diamond indicates the overall summary estimate for the analysis (width of the diamond represents the 95% CI); boxes indicate the weight of individual studies in the pooled analysis. CI: confidence interval; LVH; left ventricular hypertrophy; N-proBNP: N-terminal-pro brain natriuretic peptide; PDE5i: phosphodiesterase type 5 inhibitors.

NT-proBNP did not change (−161.9 pg/mL; 95% CI: −351.5; 27.6, *P* = 0.10) in six trials [10-14,40] (n = 407; 76% male, all treated with sildenafil or placebo). Subgroup analysis showed a significant decrease in NT-proBNP levels in patients with LVH (486.7 pg/mL; 95% CI: −712.8; −260.7, *P* <0.001) (Figure 4).

### Hemodynamic parameters

**Figure 5:**
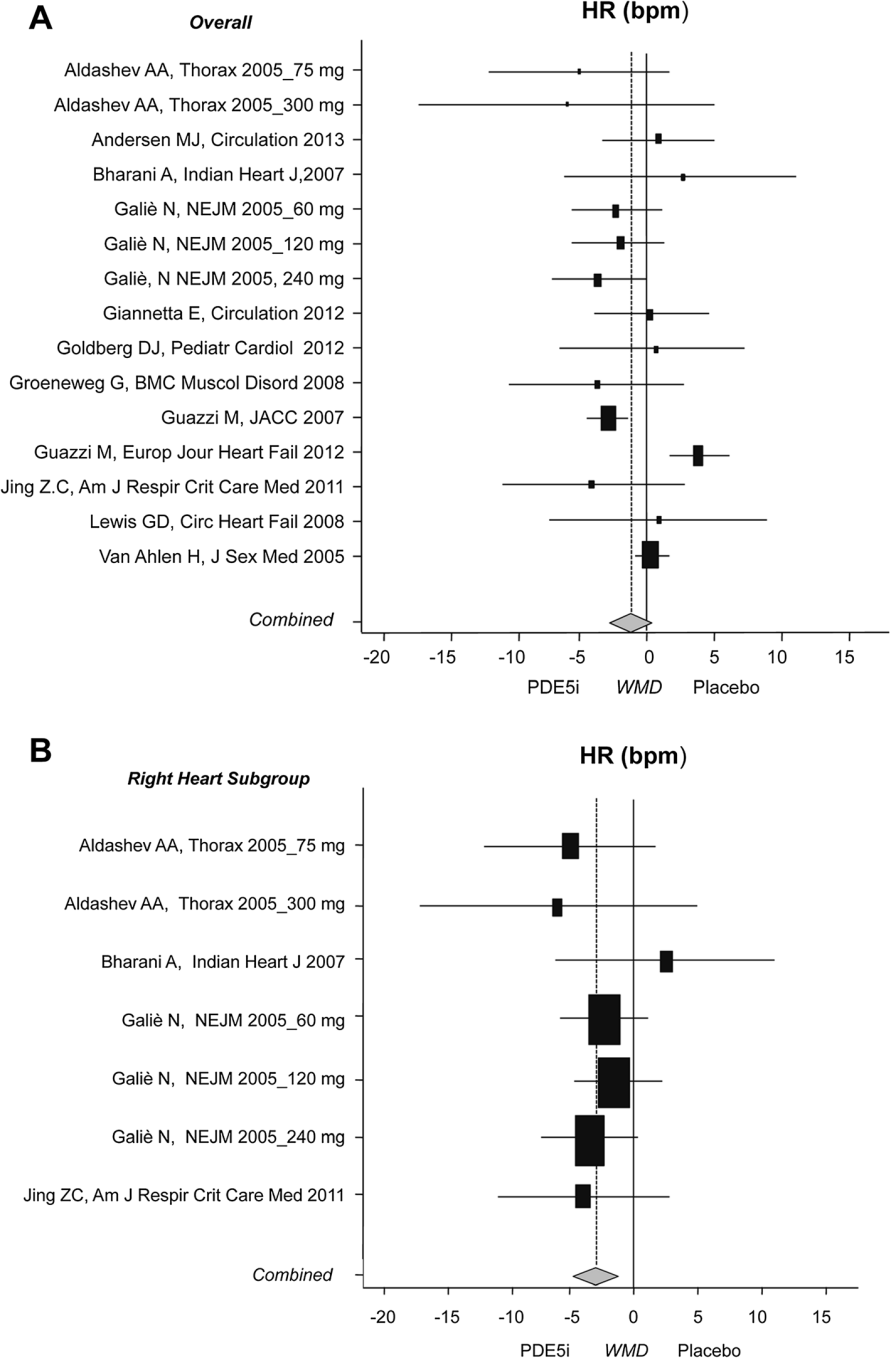
**Effects of PDE5i on heart rate. A)** Main analysis on heart rate (HR); **B)** subgroup analysis on patients with right heart disease. Diamond indicates the overall summary estimate for the analysis (width of the diamond represents the 95% CI); boxes indicate the weight of individual studies in the pooled analysis. CI: confidence interval; PDE5i: phosphodiesterase type 5 inhibitors.

PDE5i administration had no effect on HR in the main analysis (n = 977, 71% male) [2,10,11,20,24,26,28,30,31,35,37,39] (Figure 5). In the subgroup analysis a significant decrease in HR was found in patients with right heart disease (2.7 bpm; CI: −4.5; −0.8, *P* = 0.004).

In the 16 analyzed studies (n = 1,012), systolic and diastolic BP did not change (SBP; +0.616 mmHg; 95% CI: −1.113; 2.345, *P* = 0.49; DBP: −0.763 mmHg; 95% CI: −2.174; 0.648, *P* = 0.30; MAP: −0.413 mmHg; 95% CI: −3.023; 2.196, *P* = 0.80) [10-12,14,20,23-26,28,30,31,36,38,39] (see Figure 2 and Additional file 1: Figure S1). Similarly, no change was found in SVRi [10,25,30,31,39] (n = 232, 71% male) [see Additional file 1: Figure S1]. Subgroup analysis showed a significant decrease in SVRi only in patients younger than 60 (-130.44 dynes m^2^/sec cm^−5^; 95% CI: −234.44; −26.43, *P* = 0.010).

### Endothelial function

Four studies [11,24,33,34] (n = 168, all males) showed an increase in FMD (3.306%; 95% CI: 0.530; 6.082, *P* = 0.020) after PDE5i administration over placebo [see Additional file 1: Figure S2]. Subgroup analysis revealed a higher effect size in FMD of patients without cardiac disease treated with tadalafil (3.953%; 95% CI: 1.24; 6.667, *P* = 0.004). Sensitivity analysis showed a larger effect size in FMD in patients older than 60 (3.558%; 95% CI: 0.306; 6.809, *P* = 0.030).

### Safety data: PDE-5 inhibitors *versus* placebo

#### Adverse events

In our analysis, the adverse events with the highest relative risks (RRs) related to PDE5i treatment were flushing or rash (RR = 3.406; 95% CI: 1.628; 7.126, *P* = 0.001), headache (RR = 2.507; 95% CI: 1.416; 4.439, *P* = 0.002), gastric symptoms (RR = 4.138; 95% CI: 1.564; 10.946, *P* = 0.004) and epistaxis (RR = 4.701; 95% CI: 1.314; 16.812, *P* = 0.017) [see Additional file 1: Table S3]. No significant risk was observed in other reported side effects including: intestinal and musculoskeletal symptoms, nasopharyngitis, dizziness or tinnitus, visual disturbance and photosensitivity, skin irritation, insomnia, pruritus and dyspnea. The incidence of symptomatic hypotension and other serious adverse events was not statistically different between PDE5i and placebo treated groups.

## Discussion

This meta-analysis suggests for the first time that long-term daily PDE5i administration in mixed study populations: (1) exerts an anti-remodeling effect on cardiac geometry in patients with moderate-severe LVH, and (2) improves cardiac performance in all subjects with different clinical settings, with (3) no major changes in hemodynamic parameters and (4) a good safety profile. This analysis clarifies the discrepancies in previous trials and suggests that PDE5i is efficacious in cardiac protection in different stages of heart disease.

### Effect of PDE5i on cardiac geometry

The main analysis performed on all studies found no consistent changes in cardiac geometry. In the few studies performed on patients with severe LVH, PDE5i decreased LVMi (−12.207 g/m^2^; CI: −18.846 to −5.568, *P* <0.001), with the limitation being the small number of treated subjects. Nevertheless, the size of this reduction seems to be clinically relevant if compared to other anti-remodeling drugs such as ACEi (angiotensin converting enzyme inhibitors), ARBs (angiotensin II receptor blockers) or spironolactone, that have been shown to decrease LVMi, respectively, by 11.97 g/m^2^ [41], 16.68 g/m^2^ [41] and 11 g/m^2^ in patients with congestive HF [42]. Therefore, as observed in animal studies [9], our meta-analysis shows that in human LVH, chronic sildenafil improves cardiac geometry to an extent comparable to that of currently recommended anti-remodeling therapies, whereas in patients with normal or near-normal LVMi, PDE5i produced no measurable change in cardiac mass.

Of note is the high heterogeneity emerging from the main analysis (I^2^ = 96%, *P* <0.001), that improved when the studies were stratified according to the degree of cardiac hypertrophy at baseline (LVH: I^2^ = 88%, *P* = 0.003; non/mild LVH: I^2^ = 80%, *P* = 0.005). In addition, inconsistencies in the response to treatment seem related to the study duration. In the two trials lasting less than six months [10,11], LVMi apparently increased (+3.011 g/m^2^; CI −0.506 to 5.515; *P* = 0.018). Specifically, Andersen *et al*. enrolled subjects immediately after an acute myocardial infarction and the within-subject analysis showed no significant LVMi changes in either sildenafil (baseline: 93 ± 19 g/m^2^, post-treatment: 95 ± 20 g/m^2^) or placebo (baseline 93 ± 20 g/m^2^, post-treatment 91 ± 18 g/m^2^) treated subjects. However, given the opposite trends, the calculated WMD returned a positive effect. PDE5i have been shown to exert cardio-protection against post-ischemic fibrosis [6] and the post-acute setting of this study could account for the observed WMD (Figure 2). In contrast, Giannetta *et al*. enrolled type 2 diabetic patients with an increased LVMi (119.4 ± 25.7 g/m^2^), that did not change after sildenafil or placebo (respectively, −0.67 ± 5.07 g/m^2^ and -2.03 ± 7.64 g/m^2^). For all these reasons, the observed LVMi variation in this subgroup of studies could not be considered clinically relevant.

In the three studies lasting more than six months [12,14,25], LVMi decreased to a much greater extent (-8.446 g/m^2^; 95% CI −15.694 to −1.197; *P* = 0.022). The two studies by Guazzi *et al*. were performed in different populations with severe LVH due to HF of various etiologies (baseline LVMi = 166.4 ± 12.1 g/m^2^ [25] and 147.2 ± 30.2 g/m^2^ [12]) and were analyzed along with the study by Redfield *et al*. performed in non-LVH subjects (baseline LVMi = 65 g/m^2^; interquartile range (IQR): 54 to 78) [14]. Based on these findings, it is reasonable to assume that a certain duration of treatment and a degree of hypertrophy are necessary to observe a change in the cardiac mass as compared to changes in other geometric (EDV) and functional (EF) parameters, that might occur earlier. Length of treatment and degree of hypertrophy offer a clinical explanation to the apparently conflicting findings of the main analysis.

Cardiac remodeling is also measured with the volume-to-mass ratio known as the concentricity index (LVM/EDV) [43]. The main analysis of EDVi revealed high heterogeneity (I2 = 94%; *P* <0.001), but when restricting the analysis to non/mild LVH it dropped to 0% (*P* = 0.53). The apparently contradictory findings are probably explained by the significant differences in the characteristics of the enrolled study populations. The study by Guazzi *et al*. was the only study performed in subjects with ventricle dilatation and reduced EF (severe LVH heart failure); in fact, in the sensitivity analysis performed in non/mild-LVH patients, we detected a significant increase in EDVi (4.999 mL/m^2^; 95% CI: 3.286; 6.711, *P* <0.001, I^2^ of 0%). Since EDVi may increase in the final stages of heart failure (eccentric hypertrophy), but may also drop in the earlier stages of mild hypertrophy (concentric hypertrophy), we believe that it is clinically meaningful to attribute the observed EDVi change in the three homogeneous studies to improved relaxation of the ventricular wall. Notably, the LVM/EDV ratio in all of these trials was consistent with a ‘concentric thick LVH’ pattern [10,11,14], suggesting that at this earlier stage PDE5i improves myocardial wall viscoelastic properties resulting in a better diastolic load.

### Effect of PDE5i on cardiac performance

Our main analysis reveals for the first time a statistically and clinically meaningful improvement in cardiac performance, cardiac index and EF.

In our main analysis, the cardiac index improves under PDE5i with no heterogeneity between the studies (I^2^ = 0.0%), despite the differences in the baseline characteristics of the sample analyzed.

After dividing the sample according to the cardiovascular features, the subgroup analyses confirmed that PDE5i increases the cardiac index by 0.3 L/min/m^2^ in non-LVH patients. In LVH patients the increase in the cardiac index does not reach statistical significance. However, this result is probably due to the paucity of available data, with only two studies [25,40] involving a total of 62 subjects, one of which was performed in men with chronic thromboembolic pulmonary hypertension.

In the main analysis, EF also improves, but with highly significant heterogeneity (I^2^ 84%). Pooling studies according to LVH, we observed that in subjects with LVH the improvement in EF was greater and with lower heterogeneity (4.38%, CI: 2.059; 6.705, *P* <0.001; I^2^ = 79%), comparable to the average change obtained in large trials using the currently recommended therapies for HF. A recent meta-analysis [44] comparing different beta blockers in HF with reduced EF reported a WMD for EF of 4.1% (95% CI: 3.1; 4.9) *versus* placebo/standard treatment. Conversely, in non-hypertrophic subjects with preserved EF the change was not statistically significant (0.973%, CI: -1.329; 3.274, *P* <0.408; I^2^ = 0.0%). These results are consistent with the fact that the PDE5 enzyme is expressed at low levels within cardiomyocytes, until LVH hypertrophy and failure develop [45]. Our data show that only when the compensatory mechanisms evolve into changes in cardiac geometry [14,25] do the benefits of PDE5i on EF become clinically detectable.

A possible explanation for the observed heterogeneity is that PDE5 inhibitors work only when the target enzyme is overexpressed, a condition that parallels the degree of hypertrophy [see Additional file 1- *Limitations*]. In addition, the cardiac index, a more stable measure of cardiac performance, being a function of both chambers’ volumes and contractility, showed no significant heterogeneity for both the main and subgroup analyses. A recent analysis reported PDE5i-induced changes in EF in HF patients [46]. However, it omitted some relevant studies [25,31] and misclassified one trial [25], undermining the relevance of the findings. This led to incomplete data and methodological flaws when the authors attempted to perform a sub-group analysis within a group of just two studies. As EF and the cardiac index depend on cardiac contractility, hemodynamics and HR, the fact that we found no significant changes in hemodynamics and HR suggests that the improvement in cardiac performance observed in patients with left heart disease [10,11,25] is the result of a PDE5i-mediated effect on cardiac contractility, while in patients with right heart disease a dual mechanism of better pulmonary hemodynamics/oxygenation and improved inotropy is likely to occur.

One novelty of our work is its parallel analysis, for the first time, of the changes induced in cardiac geometry and performance, allowing an appraisal of the direct myocardial action of PDE5i in humans; until now, this had been considered merely secondary to indirect systemic/pulmonary hemodynamic changes.

### Neuroendocrine biomarkers

Analyses on NT-proBNP level, an indicator of myocardial stretch [47], follow the trend observed for ventricular mass and diastolic function [48,49], with a significant improvement in the subgroup of hypertrophic patients.

### Effect of PDE5i on hemodynamic parameters and endothelial function

No statistically significant chronotropic effect was found for PDE5i in the main analysis, except for the subgroup of patients with right heart disease [see Additional file 1: Figure S1], where PDE5i reduced HR, without heterogeneity (see Table 2 and Additional file 1- *Heterogeneity*). For this group, the finding could be explained in terms of better oxygenation. The chronotropic effects of PDE5i have never been investigated, but a previous human study shows that sildenafil can blunt beta-adrenergic stimulation [50] and the findings are also consistent with a general improvement in cardiac contractility [51]. A reduction of just 5 bpm in patients with HF has been associated with a lower risk of cardiovascular death and hospitalization [51], but drugs with a proven negative chronotropic effect, such as beta-blockers, produce HR reductions in the 8- to15-bpm range [51].

Regarding the effects of PDE5i on BP, our analysis shows that PDE5i given in a continuous regimen have no sustained effects on systemic BP, in a large group of disorders. This effect is confirmed by the absence of any relevant effect of PDE5i on SVRi in the main analysis. Our results confirm the findings described in men with anatomically severe coronary disease [52] where the hypothesis of a coronary steal syndrome due to PDE5i-related vasodilation was excluded using cardiac catheterism, and are consistent with those of a smaller meta-analysis [53].

Analysis of the little data available on endothelial function [11,12,34] seems to confirm a beneficial effect of continuous treatment with PDE5i, although the data were almost entirely derived from ED patients [54,55], the effects did not reach statistical significance in patients with left heart disease, and there was very high heterogeneity (I^2^ = 99%, *P* <0.001). Only the smallest [33] of the four trials analyzed is within the 95% CIs, and no relevant improvement was obtained by subgroup or sensitivity analysis [see Additional file 1-*Heterogeneity*], thus limiting the validity of the findings.

### Adverse events

Our analysis of the side effects of long-term daily use of PDE5i showed that only flushing, headache, epistaxis and gastric symptoms are statistically related to these drugs, confirming their cardiovascular safety and good tolerability. This is relevant in case future studies are planned to test PDE5i for cardiovascular disorders. It is worth noting that the majority of retrieved studies were performed in aging subjects, supporting the safety profile of these drugs when administered as a daily or continuous regimen even in this age group.

### Limitations

Our review has some limitations. The first concerns the differences in the baseline characteristics. Subgroup analyses for length of treatment (less than or more than six months) were not possible due to the small number of studies. Similarly, the majority of eligible trials provided data obtained in patients older than 60 years, using sildenafil. Furthermore, the lack of data on gender differences in cardiac outcomes did not permit the exploration of male and female behavior, whereas there have been significant advances with respect to cardiovascular disease in women in the last decade [56].

The second limitation pertains to the often missing information on cardiac geometry parameters; while considering that for all outcomes, 25% of trials had a risk of reporting bias.

The third concern is that the findings of this study are nonetheless prone to publication bias and that 13 out of 24 trials had unclear potential for selection bias (although none with a high risk), that may threaten their validity. On the positive side, industrial bias seems limited, as most trials were spontaneous studies that received only external support. The fact that some studies did not report data on all basic outcomes reinforces the value of the COMET initiative aimed at defining the minimum standardized set of outcomes that each clinical trial should report for specific health conditions [57].

A fourth concern is on the high heterogeneity found in the main analysis. In most, but not all, cases, this was reasonably explained by the inclusion criteria of the trials, degree of hypertrophy and chamber dilation, or by duration of treatment.

Finally, conclusions drawn from subgroup analyses considering only two studies are only indicative and should be interpreted with care. See specific limitations in Additional file 1-*Limitations*.

## Conclusions

With respect to the initial purpose of the study, we showed that in selected cohorts long-term continuous PDE5i administration can produce clinically meaningful improvements in cardiac remodeling and performance with an excellent cardiovascular safety and tolerability profile even in older patients and under prolonged use. The findings are based on recent RCTs that, albeit well-performed, involved small and heterogeneous populations. With these limitations, our analyses suggest that in humans, *in vivo*, PDE5i exert anti-remodeling properties that become evident only when the hypertrophic trigger results in overexpression of the targeted enzyme in cardiomyocytes. The effects are initially seen as an improvement in diastolic function (LVM/EDV ratio) and, subsequently, as a reduction in LVMi in patients with moderate to severe LVH. All the above findings occur without any relevant changes in hemodynamics, as evaluated through systemic BP and SVRi.

Our analyses reveal that the ideal target population to benefit from PDE5 is patients with HF and LVH. Given the paucity of published data, on the basis of these encouraging findings large clinical trials are urgently needed on the long-term effects of continuous PDE5i administration, focusing on cardiovascular outcomes and sex-specific response in these patients.

## Additional file

Additional file 1:
**Additional statistical analyses, heterogeneity, risk of bias, limitations, additional tables and figures.**

## Abbreviations

ACEi: angiotensin converting enzyme inhibitors
ARBs: angiotensin II receptor blockers
BP: blood pressure
bpm: beats per minute
cAMP: cyclic adenosine monophosphate
cGMP: cyclic guanosine monophosphate
CI: confidence interval
DB: double-blind
DBP: diastolic blood pressure
E/A ratio: the ratio of the early (E) to late (A) ventricular filling velocities
ED: erectile dysfunction
EDVi: end-diastolic volume index
EF: ejection fraction
FMD: flow mediated vasodilation
HF: heart failure
HR: heart rate
IQR: interquartile range
IVS: interventricular septum
LVH: left ventricular hypertrophy
LVMi: left ventricular mass index
MAP: mean arterial blood pressure
NTpro-BNP: N-terminal-pro brain natriuretic peptide
PAH: pulmonary arterial hypertension
PC: placebo-controlled
PDE1: phosphodiesterase type 1
PDE2: phosphodiesterase type 2
PDE5i: phosphodiesterase type 5 inhibitors
RCTs: randomikzed controlled trials
RR: relative risk
SBP: systolic blood pressure
SD: standard deviation
SVRi: systemic vascular resistance index
VTD: ventricular transverse diameter
Wks: weeks
WMD: weighted mean difference
