# Supplementary material for: Is chronic inhibition of phosphodiesterase type 5 cardioprotective and safe? A meta-analysis of randomized controlled trials
Source: BMC Med. 2014 Oct 20;12:185. doi: 10.1186/s12916-014-0185-3 (PMC4201993; doi:10.1186/s12916-014-0185-3)
Supplement: Additional file 1: — Additional statistical analyses, heterogeneity, risk of bias, limitations, additional tables and figures. [file 12916_2014_185_MOESM1_ESM.docx]

**Additional file 1**

1. **Statistical analyses**

Where the data were reported as medians and interquartile (IQR) ranges, corresponding means ± SDs were calculated applying the method suggested by the Cochrane Collaboration: the medians were taken as the mean values, while the SDs were calculated as IQR/1.35 (CHAPTER 7.7.3.5 Medians and interquartile ranges; Higgins JPT, Green S (editors). *Cochrane Handbook for Systematic Reviews of Interventions* Version 5.1.0 [updated March 2011]. The Cochrane Collaboration, 2011. Available from [www.cochrane-handbook.org](http://www.cochrane-handbook.org).).

Adverse events in the treatment group compared to the placebo group were analyzed by relative risks calculated on the intention-to-treat population. For continuous endpoints, the treatment effect was evaluated as the mean difference between the change from baseline values of the treatment group and the placebo group with 95% CIs [Morris SB, 2008. Estimating Effect Sizes From Pretest-Posttest-Control Group Designs. Organizational Research Methods, 11(2):364-386].

The mathematical formula is:


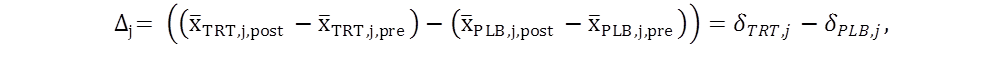


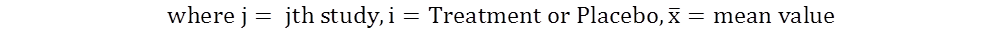


As previously mentioned, this was calculated if unavailable in published papers.

A more detailed explanation is necessary for the calculation of the standard deviations. The formula reads:


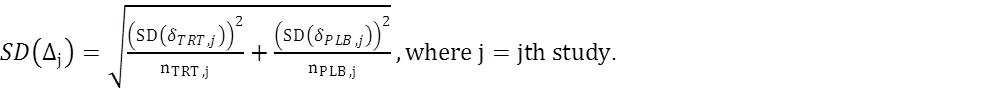


Firstly, the standard deviation of the change from baseline values was calculated using:


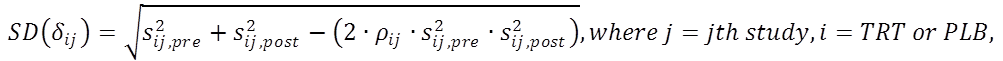


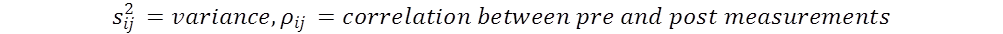


The variances for pre- and post- treatment/placebo were known, while the degree of correlation was always unknown and had to be guessed. When for a given outcome, δ_ij_ and the corresponding SD (δ_ij_) were available for one or more studies, ρ_ij_ was calculated rearranging the formula for SD (δ_ij_) and the average degree of correlation was used as an ‘educated’ guess for the jth study for which SD (δ_ij_)was unknown. On the other hand, when no study reported δ_ij_ and the corresponding SD (δ_ij_), a ρ_ij_ of 0.90 was hypothesized.

Once SD (δ_ij_) for the treatment and the placebo groups had been calculated for each study on each outcome, the SD for the treatment effect SD (Δ_j_) could be calculated using the formula specified above.

A meta-analysis was performed on all outcomes and effect sizes were combined to give a pooled estimate of a weighted average of the treatment effects Δ_j_ for each study, the weights being the reciprocals of the variance**.**

1. **Heterogeneity**

As already mentioned in the main body of the article, the studies included in this review were very heterogeneous in the population characteristics. Starting from the main analysis, we explored the pattern of heterogeneity in all subgroup analyses, or sensitivity analyses, performed for all outcomes. Results are herein reported. Cut-offs for the I^2^ statistic were: low = 30%; moderate = 30‑75%; high ≥75%.

**2.1 Cardiac geometry**

**LVMi** – High heterogeneity emerged from the main analysis (I^2^ = 96%, p <0.001), and an improvement was found when stratifying the analysis by hypertrophy (LVH: I^2^  = 88%, p = 0.003; non/mild LVH: I^2^  = 80%, p = 0.005). Heterogeneity was high when restricting the analysis to age >60 years (I^2^  = 95%, p <0.001).

**EDVi** – The main analysis on this outcome showed a high heterogeneity with I^2^ 94% (p <0.001), but when restricting the analysis to the non/mild LVH subgroup this dropped to 0% (p = 0.53).

**IVS and VTD –** The main analysis on these outcomes included only 2 widely varying studies (I^2^  = 95%, p <0.001 and I^2^  = 90%, p <0.001, respectively).

**2.2 Cardiac performance**

**Cardiac Index –** All studies reported similar findings for this outcome and an I^2^ of 0% was calculated from the main analysis (p = 0.66).

**EF –** From the main analysis an I^2^ of 84% was calculated and the test for heterogeneity was highly statistically significant (p <0.001). This remained high for LVH (I^2^  = 79%, p = 0.008), while for non/mild-LVH it dropped to 0% (p = 0.19). High heterogeneity was also observed in the age subgroups (<60 years: I^2^  = 80%, p = 0.024; >60 years: I^2^ = 90%, p <0.001).

**E/A ratio –** The main analysis for this outcome showed very high heterogeneity (I^2^ = 93%, p = 0.753). p <0.001). No improvement was obtained when the analysis was restricted to left-heart disease (I^2^ = 96%, p <0.001) and to <60 years of age (I^2^ = 90%, p = 0.001).

**2.3 Cardiac biomarkers**

**NT-proBNP** – The high heterogeneity that emerged from the main analysis on this outcome (I^2^ = 82%, p <0.001) was annulled in the LVH subgroup (I^2^ = 0%, p = 0.52), but was moderate in the non/mild LVH group (I^2^ = 75%, p = 0.017). Restricting the analysis to left heart disease and to >60 years, heterogeneity remained moderate-high (I^2^ = 84%, p <0.001, and I^2^ = 72%, p = 0.013, respectively).

**2.4 Hemodynamic parameters**

**HR –** From the main analysis a moderate heterogeneity emerged for this outcome (I^2^ = 56%, p = 0.004); this could be removed by stratifying to LVH and non/mild LVH (I^2^ = 0.0%, p = 0.96 for both groups). The heterogeneity was also removed by restricting the analysis to HF patients with reduced EF (I^2^ = 0.0%, p = 0.38). In the right heart disease group the heterogeneity was also 0% (p = 0.85). In the left-heart disease it remained high (I^2^ = 81%, p <0.001). From the subgroup analysis by drug it emerged that heterogeneity remained moderate in the sildenafil group (I^2^ = 64%, p = 0.002), and was removed in the vardenafil and tadalafil groups (I^2^ = 0.0%, p = 0.23, and I^2^ = 0.0%, p = 0.27 respectively). Heterogeneity was removed in the <60 years group (I^2^ = 0.0%, p = 0.86), but increased to 79% (p <0.001) in the >60 years group. Lastly, the analysis was restricted to the non-cardiac disease group and an I^2^ = 0.0% (p = 0.27) was estimated.

**SBP –** Moderate heterogeneity emerged for this outcome (I^2^ = 49%, p = 0.031), which dropped to 0.0% in the LVH group (p = 0.90) and increased to 79% in the non/mild LVH group (p = 0.007). Increased heterogeneity in the non/mild LVH group could be related to the inclusion of patients with cardiac and non-cardiac diseases. No improvement was observed on restricting the analysis to left-heart disease, (I^2^ = 66%, p = 0.007).

Heterogeneity remained moderate (I^2^ = 60%, p = 0.013) in the sildenafil subgroup and was removed in the tadalafil group (I^2^ = 0.0%, p = 0.69). There was no heterogeneity in the <60 years group (I^2^ = 0.0%, p = 0.43) while it was moderate in the >60 years group (I^2^ = 72%, p = 0.006). Lastly, in the non-cardiac disease group heterogeneity was absent (I^2^ = 0.0%, p = 0.50).

**DBP –** The main analysis of this outcome also revealed moderate heterogeneity (I^2^ = 52%, p = 0.018). Stratifying the analysis by LVH did not make any significant improvement (non/mild LVH: I^2^ = 91%, p = 0.001, and LVH: I^2^ = 66%, p = 0.05). Heterogeneity was removed in the right-heart disease group (I^2^ = 0.0%, p = 0.99), but increased in the left-heart disease group (I^2^ = 75%, p = 0.001). When considering the treatments, heterogeneity remained moderate in the sildenafil group (I^2^ = 63%, p = 0.006), but was removed in the tadalafil group (I^2^ = 0.0%, p = 0.78). No heterogeneity emerged in the <60 years group (I^2^ = 0.0%, p = 0.89), but there was higher heterogeneity in the >60 years group compared to the main analysis (I^2^ = 71%, p = 0.007). No heterogeneity was found in the non-cardiac disease group (I^2^ = 0.0%, p = 0.65).

**MAP –** The main analysis performed on this outcome found moderate heterogeneity (I^2^ = 71%, p = 0.002), which could be removed when restricting the analysis to the LVH group (I^2^ = 0.0%, p = 0.24). An analysis restricted to the left-heart disease group did not reduce heterogeneity (I^2^ = 75%, p = 0.002), nor did analysis of the sildenafil group only (I^2^ = 72%, p = 0.003). When stratifying by age, no heterogeneity was found in the <60 years group (I^2^ = 0.0%, p = 0.67), but there was high heterogeneity in the >60 years group (I^2^ = 83%, p = 0.002).

**SVRi –** High heterogeneity was found among all studies reporting findings on this outcome (I^2^ = 89%, p <0.001). Subgroup and sensitivity analyses brought some improvement in the <60 years group only (I^2^ 0.0%, p = 0.014).

**2.5 Endothelial Function**

**FMD** – Very high heterogeneity was found in the main analysis (I^2^ = 99%, p <0.001) and no significant improvement was obtained by any subgroup or sensitivity analysis. In both the left-heart disease group and <60 years groups the I^2^ was 99% (p <0.001), and in the non-cardiac disease group it was 96% (p <0.001), thus limiting the validity of the findings.

**2.6 Adverse Events**

No heterogeneity was found among studies which reported the following adverse events: tinnitus, dyspnea, skin irritation, and atrial fibrillation. For all other adverse events a high level of heterogeneity was found, but this was statistically significant only for the following: gastric disorders (dyspepsia, pyrexia, gastritis), flushing or rash, musculoskeletal disorders (pain in limb, back pain, myalgia, muscle cramps), headache, and pruritus.

1. **Risk of bias**

All publications reported results from RCTs. However, the randomization method and allocation concealment were inappropriately described in 54% of studies, so the risk of selection bias was unclear for this group, while for the remaining 46% of trials the risk of selection bias was low.

All studies were of low risk for performance and detection bias.

A low risk of reporting bias was allocated to 62.5% of studies. One study ^1^ did not have enough information, so the risk for this bias was unclear, while 37.5% were judged to have a medium-high risk of reporting bias due to incomplete reporting of some outcomes of interest (only baseline scores reported, or scores reported in graphs, but not in tables); some outcomes were measured but not reported; some outcomes were reported as indexed scores at baseline but not post-treatment.

A medium-high risk for other biases was allocated to some RCTs in which enrollment of the same population was suspected ^2-5^, or the sample was widely heterogeneous ^6^, or the study design was a crossover without washout period.

**4. Limitations**

We observed specific limitations in subgroup analyses due to the coupling of studies and the paucity of available data.

*Cardiac geometry*

For LVMi, the subgroup for length of treatment revealed conflicting results (data not shown). In the two studies ^10;11^ lasting less than 6 months, LVMi increased significantly (+3.011 g/m^2^; CI -0.506 to 5.515; p = 0.018). Andersen et al. included subjects after acute myocardial infarction, in which normal baseline LVMi did not significantly increase after sildenafil (from 93±19 g/m^2^ to 95±20 g/m^2^) whereas in the placebo group LVMi did not decrease significantly (from 93±20 g/m^2^ to 91±18 g/m^2^). Giannetta et al. performed an RCT on type 2 diabetic patients in which elevated LVMi (119.4±25.7 g/m^2^) did not significantly decrease after sildenafil and placebo (respectively, ‑0.67±5.07 g/m^2^ and -2.03±7.64g/m^2^). Notably, the observed LVMi increase in the subgroup analysis was not clinically significant.

In the three studies ^6-8^ lasting more than 6 months, LVMi decreased significantly and clinically (‑8.446 g/m^2^; 95% CI -15.694 to -1.197; p = 0.022). The two studies by Guazzi M. et al. were performed in patients with LVH due to HF of various etiologies (baseline LVMi = 166.4±12.1 g/m^2^ – ^8^ and 147.2±30.2 g/m^2^ ^7^) and were analyzed together with the study of Redfield M. et al ^6^ that was conducted in non-LVH subjects (baseline LVMi = 65 g/m^2^; IQR: 54-78).

The studies analyzing IVS and VTD were widely different from each other and the heterogeneity is likely due to the paucity of available data. Only two studies were available for IVS, performed in type 2 diabetic men (Giannetta et al.) and in men with severe cardiac hypertrophy (Guazzi et al. Circulation) for a total of 98 subjects. For VTD also, only two studies could be analyzed, performed in type 2 diabetic men (Giannetta) and in men with severe cardiac hypertrophy (Guazzi Circ heart failure) for a total of 99 subjects.

*Cardiac performance*

The analysis of EF showed a high heterogeneity (I^2^ 84%), probably due to the baseline clinical features of the sample analyzed. Pooling studies according to LVH, we observed that in non-hypertrophic subjects with preserved EF the change was not statistically significant (0.973%, CI: -1.329 to 3.274, p <0.408) but heterogeneity dropped to 0.0%. It is therefore reasonable to assume that the degree of chamber geometry offers a clinical explanation of the heterogeneity.

However, in subgroup analysis for subjects with LVH we observed that the improvement in EF was greater (4.38%, CI: 2.059 to 6.705, p <0.001) and heterogeneity slightly decreased (I^2^ = 79%) with respect to the main analysis. The subgroup analysis for LVH patients included three studies. Specifically, Guazzi et al. (Circulation 2011) enrolled subjects with right ventricle failure and PAH with preserved EF and the analysis showed that EF increased significantly (p <0.01) after 12 months of sildenafil treatment (baseline: 60±4% post-treatment: 63±3%) compared to placebo (baseline 60±6% post-treatment 68±7%).

In contrast, Guazzi et al. (JACC 2007) enrolled patients with chronic HF and reduced performance, in which EF did not significantly change after 6 months’ treatment with sildenafil (baseline: 30.6±3% post-treatment: 34.7±2.8%) or placebo (baseline: 31.9±3.3% post-treatment: 30.4±3.6%).

Furthermore, in a study of patients with systolic heart failure and reduced EF Guazzi et al. (Circ. Heart Fail. 2011) found a significant EF increase after 12 months of sildenafil administration (baseline: 29.5±3.0% post-treatment: 36.3±3.0%, p <0.01) in the within-subject analysis and compared to placebo (p <0.01). The heterogeneity was thus not explained by reduced or preserved performance status.

To better comprehend the EF heterogeneity we performed a subgroup analysis for patients with reduced EF (<35%, according to the ESC Guidelines for the diagnosis and treatment of acute and chronic heart failure 2012). The subgroup analysis produced a significant increase in EF (4.8%, CI: 2.989 to 6.633, p <0.001) but confirmed the high heterogeneity (I^2^ = 79%), suggesting that performance status alone did not explain the heterogeneity.

As the main and subgroup analyses on Cardiac Index did not suffer from heterogeneity (I^2^ = 0.0%) and the EF and Cardiac Index depend on cardiac chambers, the fact that we found high heterogeneity in the EF analysis without heterogeneity in the Cardiac Index analysis could suggest that the variability of EF measurements may be responsible for this observation.

Due to the availability of the data, we performed the subgroup analysis for reduced performance status (EF<35%) for EF only.

The analysis of E/A ratio showed no changes, probably due to the paucity of data, the heterogeneity of the clinical conditions of the patients enrolled in the trials ^3;7;8^ and because the baseline values were normal according to age (E/A ratio = 1.88±0.45; CI: 0.98 to 2.78, from 16 to 20 years; 0.96 ± 0.18; CI: 0.6 to 1.32, over 60 years) ^9^.

*Additional File1- Table1*. **Characteristics of studies: No. patients (ITT), No.**

**males *vs.* females. S, sildenafil; T, tadalafil; V, vardenafil; P, placebo.**

| **Study** | **No. Patients (ITT)** | **No. Males *vs*. Fe males** |
| --- | --- | --- |
| **Aldashev AA, *Thorax*, 2005 ^12^** | 9 S *vs*. 8 P  5 S *vs*. 8 P | All male |
| **Amin A, *Congest Heart Fail*, 2013 ^13^** | 53 S *vs*. 53 P | S: 38 m *vs.* 15 f  P: 40 m *vs.* 13 f |
| **Andersen MJ, *Circulation*, 2013 ^10^** | 35 S *vs*. 35 P | S: 32 m *vs.* 3 f  P: 32 m *vs.* 3 f |
| **Badesch, *J Rheumatol*, 2007 ^14^** | 21 S  20 S  21 S  22 P | S: 5 m *vs.* 16 f  3 m *vs.* 17 f  2 m *vs.* 19 f  P: 4 m *vs.* 18 f |
| **Behling A, *J of Cardiac Fail*, 2008 ^15^** | 11 S *vs*. 8 P | S: 9 m *vs.* 2 f  P: 4 m *vs.* 4 f |
| **Bharani A, *Indian Heart J*, 2007 ^16^** | 8 T – P | T-P: 4 m *vs.* 4 f |
| **Bocchio M, *Atherosclerosis*, 2008 ^17^** | 18 T *vs*. 18 P | All male |
| **Galiè N, *NEJM*, 2005 ^18^** | 69 S  68 S  71 S  70 P | S: 20 m *vs.* 49 f  21 m *vs.* 47 f  15 m *vs.* 56 f  P: 13 m *vs.* 57 f |
| **Giannetta E, *Circulation*, 2012 ^11^** | 29 S *vs*. 25 P | All male |
| **Goldberg DJ, *Circulation*, 2011 ^2^** | 28 S - P | S-P: 18 m *vs.* 10 f |
| **Goldberg, *Pediatr Cardiol*, 2012 ^3^** | 28 S - P | S-P: 18 m *vs.* 10 f |
| **Groeneweg G, BMC *Muscoloskeletan disorders*, 2008 ^1^** | 12 T *vs*. 12 P | T: 3 m *vs.* 9 f  P: 1 m *vs.* 11 f |
| **Guazzi M, *J* *Am Coll Cardiol*, 2007 ^19^** | 23 S *vs*. 23 P | All male |
| **Guazzi M, *Circulation*, 2011 ^8^** | 22 S *vs*. 22 P | S: 17 m *vs.* 5 f  P: 18 m *vs.* 4 f |
| **Guazzi M, *Circ Heart Fail,* 2011 ^7^** | 23 S *vs*. 22 P | All male |
| **Guazzi M, *Europ J Heart* Failure, 2012 ^20^** | 16 S *vs*. 16 P | All male |
| **Jing, Z.C, *Am J Resp Crit Care Med*, 2011 ^21^** | 43 V *vs.* 16 P | V: 8 m *vs.* 35 f  P: 2 m *vs.* 14 f |
| **Lewis GD, *Circulation*, 2007^4^** | 17 S *vs*. 17 P | S: 14 m *vs.* 3 f  P: 15 m *vs.* 2 f |
| **Lewis GD, *Circ Heart Fail,* 2008 ^4;5^** | 15 S *vs*. 15 P | S&P: 27 m *vs.* 3 f |
| **Rosano G MC , *European Urology*, 2005 ^22^** | 16 T *vs*. 16 P | All male |
| **Redfield MM, *JAMA,* 2013 ^6^** | 113 S *vs*. 103 P | S: 64 m *vs.* 49 f  P: 48 m *vs.* 55 f |
| **Sastry BKS, *JACC,* 2004 ^23^** | 32 S - P | S-P: 20 m *vs.*12 f |
| **Suntharalingam J, *Chest,* 2008 ^24^** | 9 S *vs*. 10 P | S: 2 m *vs.* 7 f  P: 7 m *vs.* 3 f |
| **Van AH, *J Sex Med ,*2005 ^25^** | 175 V *vs*. 175 P | All male |

*Additional File 1- Table 2.* **Risk of bias summary per Cochrane metrics. '+' indicates present**

| STUDY | SELECTION BIAS | PERFORMANCE  BIAS | DETECTION  BIAS | ATTRITION  BIAS | REPORTING  BIAS | OTHER  BIAS |
| --- | --- | --- | --- | --- | --- | --- |
| Aldashev AA, *Thorax*, 2005 ^12^ | **?** | **-** | **-** | **-** | **-** | **-** |
| Amin A, *Congest Heart Fail*, 2013 ^13^ | **-** | **-** | **-** | **-** | **+** | **-** |
| Andersen MJ, *Circulation*, 2013 ^10^ | **-** | **-** | **-** | **-** | **-** | **-** |
| Badesch, *J Rheumatol*, 2007 ^14^ | **-** | **-** | **-** | **-** | **-** | **-** |
| Behling A, *J of Cardiac Fail*, 2008 ^15^ | **?** | **-** | **-** | **-** | **+** | **-** |
| Bharani A, *Indian Heart J*, 2007 ^16^ | **?** | **-** | **-** | **-** | **-** | **-** |
| Bocchio M, *Atherosclerosis*, 2008 ^17^ | **-** | **-** | **-** | **-** | **+** | **-** |
| Galiè N, *NEJM*, 2005 ^18^ | **?** | **-** | **-** | **-** | **-** | **-** |
| Giannetta E, *Circulation*, 2012 ^11^ | **-** | **-** | **-** | **-** | **-** | **-** |
| Goldberg DJ, *Circulation*, 2011 ^2^ | **?** | **-** | **-** | **-** | **-** | **-** |
| Goldberg, *Pediatr Cardiol*, 2012 ^3^ | **?** | **-** | **-** | **-** | **+** | **+** |
| Groeneweg G, BMC *Muscoloskeletan disorders*, 2008 ^1^ | **-** | **-** | **-** | **-** | **?** | **-** |
| Guazzi M, *J* *Am Coll Cardiol*, 2007 ^19^ | **-** | **-** | **-** | **-** | **-** | **-** |
| Guazzi M, *Circulation*, 2011 ^8^ | **-** | **-** | **-** | **-** | **-** | **-** |
| Guazzi M, *Circ Heart Fail,* 2011 ^7^ | **-** | **-** | **-** | **-** | **+** | **-** |
| Guazzi M, *Europ J Heart* Failure, 2012 ^20^ | **-** | **-** | **-** | **-** | **-** | **-** |
| Jing, Z.C, *Am J Resp Crit Care Med*, 2011 ^21^ | **-** | **-** | **-** | **-** | **-** | **-** |
| Lewis GD, *Circulation*, 2007 ^4^ | **?** | **-** | **-** | **-** | **+** | **-** |
| Lewis GD, *Circ Heart Fail,* 2008 ^5^ | **?** | **-** | **-** | **-** | **-** | **+** |
| Rosano G MC , *European Urology*, 2005 ^22^ | **?** | **-** | **-** | **-** | **-** | **-** |
| Redfield MM, *JAMA,* 2013 ^6^ | **?** | **-** | **-** | **-** | **?** | **-** |
| Sastry BKS, *JACC,* 2004 ^23^ | **?** | **-** | **-** | **-** | **-** | **+** |
| Suntharalingam J, *Chest,* 2008 ^24^ | **?** | **-** | **-** | **-** | **-** | **-** |
| Van Ahlen H, J Sex Med, 2005 ^25^ (la voce giusta è 36 | **?** | **-** | **-** | **-** | **+** | **-** |

*Additional File1-Table3***. Summary of findings on Adverse Events (AEs).**

| **Adverse events** | **No.**  **Patients**  **(ITT)**  **PDE5i *vs.* Plb** | **RR**  **95% CI** | ***p value*** | **I-square**  **[het. b/w studies]** | **References** |
| --- | --- | --- | --- | --- | --- |
| **Flushing or Rash** | 1217  731 *vs.* 514 ‡ | 3.406  [1.628; 7.126] | ***0.001*** | 85.83% | ^2;6;13-15;18-21;25^ |
| **Headache** | 1260  768 *vs.* 552‡ | 2.507  [1.416; 4.439] | ***0.002*** | 80.19% | ^2;6;10;13-15;18;21;23-25^ |
| **Gastric**  **(Dyspepsia, Pyrexia, Gastritis)** | 588  382 *vs.* 206 | 4.138  [1.564;10.946] | ***0.004*** | 92.16% | ^10;13;14;18;20;24^ |
| **Epistaxis** | 467  322 *vs.* 145 | 4.701  [1.314;16.812] | ***0.017*** | 89.59% | ^13;14;18^ |
| **Insomnia** | 527  366 *vs.* 161 | 2.760  [0.946; 8.052] | *0.063* | 85.58% | ^13;14;18;21^ |
| **Musculoskeletal**  **(pain in limb, back pain, myalgia, muscle cramps)** | 554  302 *vs.* 252‡ | 2.622  [0.822; 8.370] | *0.103* | 95.07% | ^2;13;14;18;21^ |
| **Visual**  **(Photosensitivity, visual disturbance)** | 343  220 vs. 151‡ | 2.303  [0.650; 8.157] | *0.196* | 90.60% | ^2;13;18^  ~ |
| **Symptomatic hypotension** | 244  141 *vs.* 131‡ | 2.088  [0.093;47.072] | *0.643* | 99.39% | ^2;6^ |
| **Pruritus** | 190  115 *vs.* 75 | 1.564  [0.068;35.967] | *0.780* | 99.48% | ^13;14^ |
| **Dyspnea** | 286  148 vs. 138 | 1.477  [0 .710; 3.069] | *0.296* | 0% | ^6;10^ |
| **Nasopharyngitis** | 190  115 *vs.* 75 | 1.474  [0.271; 8.002] | *0.653* | 98.03% | ^13;14^ |
| **Intestinal**  **(diarrhea, abdominal pain)** | 586  409 *vs.* 205‡ | 1.368  [0.558; 3.353] | *0.493* | 88.06% | ^2;13;14;18;20;21^ |
| **Skin irritation** | 305  157 *vs.* 148 | 0.929  [0.441; 1.959] | *0.847* | 0% | ^6;10;24^ |
| **Dizziness or tinnitus** | 428  248 *vs.* 208‡ | 0.910  [0.516; 1.607] | *0.746* | 0% | ^2;6;13;15;21^ |
| **Death** | 301  215 *vs.* 118‡ | 0.658  [0.133; 3.252] | *0.608* | 90.03% | ^18;21;23^  ~~ |
| **Atrial fibrillation** | 78  39 *vs.* 39 | 0.280  [0.049; 1.604] | *0.153* | 0% | ^19;20^ |

‡ : inclusion of studies with a cross over design; ~ : Galiè et al. 120 mg/ die and 240 mg/die; ~~Galiè et al. 60 mg/die and 240 mg

**Additional File 1. FIGURES**

**Figure 1. Effects of PDE5i over Placebo on other hemodynamic parameters.**

A, Main analysis on MAP; B; main analysis on SVRi. Diamond indicates the overall summary estimate for the analysis (width of the diamond represents the 95% CI); boxes indicate the weight of individual studies in the pooled analysis.

A

B

**Figure 2. Effects of PDE5i over Placebo on flow mediated vasodilation.**

FMD: main analysis. Diamond indicates the overall summary estimate for the analysis (width of the diamond represents the 95% CI); boxes indicate the weight of individual studies in the pooled analysis.

****Reference List

(1) Groeneweg G, Huygen FJ, Niehof SP et al. Effect of tadalafil on blood flow, pain, and function in chronic cold complex regional pain syndrome: a randomized controlled trial. BMC Musculoskelet Disord 2008;9:143.

(2) Goldberg DJ, French B, McBride MG et al. Impact of oral sildenafil on exercise performance in children and young adults after the fontan operation: a randomized, double-blind, placebo-controlled, crossover trial. Circulation 2011;123:1185-1193.

(3) Goldberg DJ, French B, Szwast AL et al. Impact of sildenafil on echocardiographic indices of myocardial performance after the Fontan operation. Pediatr Cardiol 2012;33:689-696.

(4) Lewis GD, Shah R, Shahzad K et al. Sildenafil improves exercise capacity and quality of life in patients with systolic heart failure and secondary pulmonary hypertension. Circulation 2007;116:1555-1562.

(5) Lewis GD, Shah RV, Pappagianopolas PP, Systrom DM, Semigran MJ. Determinants of ventilatory efficiency in heart failure: the role of right ventricular performance and pulmonary vascular tone. Circ Heart Fail 2008;1:227-233.

(6) Redfield MM, Chen HH, Borlaug BA et al. Effect of phosphodiesterase-5 inhibition on exercise capacity and clinical status in heart failure with preserved ejection fraction: a randomized clinical trial. JAMA 2013;309:1268-1277.

(7) Guazzi M, Vicenzi M, Arena R, Guazzi MD. PDE5 inhibition with sildenafil improves left ventricular diastolic function, cardiac geometry, and clinical status in patients with stable systolic heart failure: results of a 1-year, prospective, randomized, placebo-controlled study. Circ Heart Fail 2011;4:8-17.

(8) Guazzi M, Vicenzi M, Arena R, Guazzi MD. Pulmonary hypertension in heart failure with preserved ejection fraction: a target of phosphodiesterase-5 inhibition in a 1-year study. Circulation 2011;124:164-174.

(9) Nagueh SF, Appleton CP, Gillebert TC et al. Recommendations for the evaluation of left ventricular diastolic function by echocardiography. Eur J Echocardiogr 2009;10:165-193.

(10) Andersen MJ, Ersboll M, Axelsson A et al. Sildenafil and diastolic dysfunction after acute myocardial infarction in patients with preserved ejection fraction: the Sildenafil and Diastolic Dysfunction After Acute Myocardial Infarction (SIDAMI) trial. Circulation 2013;127:1200-1208.

(11) Giannetta E, Isidori AM, Galea N et al. Chronic Inhibition of cGMP phosphodiesterase 5A improves diabetic cardiomyopathy: a randomized, controlled clinical trial using magnetic resonance imaging with myocardial tagging. Circulation 2012;125:2323-2333.

(12) Aldashev AA, Kojonazarov BK, Amatov TA et al. Phosphodiesterase type 5 and high altitude pulmonary hypertension. Thorax 2005;60:683-687.

(13) Amin A, Mahmoudi E, Navid H, Chitsazan M. Is chronic sildenafil therapy safe and clinically beneficial in patients with systolic heart failure? Congest Heart Fail 2013;19:99-103.

(14) Badesch DB, Hill NS, Burgess G et al. Sildenafil for pulmonary arterial hypertension associated with connective tissue disease. J Rheumatol 2007;34:2417-2422.

(15) Behling A, Rohde LE, Colombo FC, Goldraich LA, Stein R, Clausell N. Effects of 5'-phosphodiesterase four-week long inhibition with sildenafil in patients with chronic heart failure: a double-blind, placebo-controlled clinical trial. J Card Fail 2008;14:189-197.

(16) Bharani A, Patel A, Saraf J, Jain A, Mehrotra S, Lunia B. Efficacy and safety of PDE-5 inhibitor tadalafil in pulmonary arterial hypertension. Indian Heart J 2007;59:323-328.

(17) Bocchio M, Pelliccione F, Passaquale G et al. Inhibition of phosphodiesterase type 5 with tadalafil is associated to an improved activity of circulating angiogenic cells in men with cardiovascular risk factors and erectile dysfunction. Atherosclerosis 2008;196:313-319.

(18) Galie N, Ghofrani HA, Torbicki A et al. Sildenafil citrate therapy for pulmonary arterial hypertension. N Engl J Med 2005;353:2148-2157.

(19) Guazzi M, Samaja M, Arena R, Vicenzi M, Guazzi MD. Long-term use of sildenafil in the therapeutic management of heart failure. J Am Coll Cardiol 2007;50:2136-2144.

(20) Guazzi M, Vicenzi M, Arena R. Phosphodiesterase 5 inhibition with sildenafil reverses exercise oscillatory breathing in chronic heart failure: a long-term cardiopulmonary exercise testing placebo-controlled study. Eur J Heart Fail 2012;14:82-90.

(21) Jing ZC, Yu ZX, Shen JY et al. Vardenafil in pulmonary arterial hypertension: a randomized, double-blind, placebo-controlled study. Am J Respir Crit Care Med 2011;183:1723-1729.

(22) Rosano GM, Aversa A, Vitale C, Fabbri A, Fini M, Spera G. Chronic treatment with tadalafil improves endothelial function in men with increased cardiovascular risk. Eur Urol 2005;47:214-220.

(23) Sastry BK, Narasimhan C, Reddy NK, Raju BS. Clinical efficacy of sildenafil in primary pulmonary hypertension: a randomized, placebo-controlled, double-blind, crossover study. J Am Coll Cardiol 2004;43:1149-1153.

(24) Suntharalingam J, Treacy CM, Doughty NJ et al. Long-term use of sildenafil in inoperable chronic thromboembolic pulmonary hypertension. Chest 2008;134:229-236.

(25) van AH, Wahle K, Kupper W, Yassin A, Reblin T, Neureither M. Safety and efficacy of vardenafil, a selective phosphodiesterase 5 inhibitor, in patients with erectile dysfunction and arterial hypertension treated with multiple antihypertensives. J Sex Med 2005;2:856-864.
